# Introduced Western Honeybees Dramatically Reduce the Abundance of Wild Bees in Alpine Meadows, Eastern Tibet Plateau

**DOI:** 10.3390/biology14091186

**Published:** 2025-09-03

**Authors:** Ruimin An, Shucun Sun

**Affiliations:** Department of Biology, School of Life Sciences, Nanjing University, Nanjing 210093, China; dz1930006@smail.nju.edu.cn

**Keywords:** alpine meadow, *Apis mellifera*, native bee, niche overlap, plant–pollinator interactions, pollination network, rare species, Tibetan Plateau

## Abstract

Over the past few decades, the western honeybee (*Apis mellifera*) has been widely introduced throughout China. Introduced honeybees have often been observed to pose a significant threat to native bee species diversity through competition for floral resources. However, the specific impact on native pollinator communities is not well understood, especially in alpine meadows, where bee diversity is particularly high. In this study, we determined the difference in species abundance and diversity of native bees between nearby and distant plots relative to apiaries in alpine meadows on the eastern Tibetan Plateau. We constructed a plant–bee network and calculated perceived apparent competition (i.e., the feeding niche overlap) between the introduced honeybees and each of the native bee species. Furthermore, we determined the relationship between the relative change in species abundance and the perceived apparent competition and species abundance across bee species. Among the 15 native bee species, 9 bee species were significantly lower in abundance in the nearby plots compared to the distant plots, and, in particular,, 5 rare species were not found in the nearby plots. For the other six species, the abundance difference was statistically non-significant. Data analysis reveals that the species abundance of native bees in distant plots, along with the feeding niche overlap between introduced bees and native bees in distant plots, explains the variation in the relative change in species abundance across bee species. However, the feeding niche overlap between introduced bees and native bees in nearby plots does not account for this variation. Our findings demonstrate that rare native bee species with greater feeding niche overlap have been significantly impacted by the introduced western honeybees. These results highlight important implications for pollinator management in natural ecosystems and the conservation of wild bees.

## Simple Summary

Western honeybees have been introduced across China, yet their effects on native pollinators in alpine meadows remain unclear. We compared native bee abundance and diversity close to and far from apiaries on the eastern Tibetan Plateau, analyzing plant–bee networks and niche overlap (apparent competition) between honeybees and native species. The results show that native bee abundance in distant plots and their niche overlap with honeybees explain interspecific variations in species abundance changes.

## 1. Introduction

The western honeybee (*Apis mellifera*) is one of the earliest domesticated insects. It is the primary species maintained by beekeepers worldwide for its honey production and pollination services [1]. Through human assistance, the western honeybee has been introduced to every continent except Antarctica [2]. It was introduced to China in the late 19th century and has since become a common species in beekeeping. Several subspecies, such as *Apis mellifera ligustica* and *A. mellifera carnica*, are prevalent. Currently, China has approximately 6.8 million colonies of western honeybees and over three hundred thousand beekeepers. Beekeepers migrate hives from southern to northern regions and from lowland to high-altitude areas across the country [3,4]. While providing pollination services to crops and producing honey and other hive products for commercial markets, the western honeybee also has negative impacts on wild pollinators [5,6].

As a eusocial insect, the western honeybee is recognized as a highly impactful invasive species [7]. This is due to its ability to rapidly exploit resources, such as nectar and pollen, with a coordinated group of foragers in its introduced range. This exploitation leads to significant ecological impacts on wild pollinators through competition for food resources in natural ecosystems [5]. For instance, in North America, the introduction of *Apis mellifera* has caused intense competition with native bumblebees (*Bombus* spp.), resulting in population declines as resources are depleted for native bee species [8]. Additionally, the introduced honeybees can have indirect effects on native pollinators. In South America, for example, the presence of *Apis mellifera* has altered the pollination dynamics of native plants, favoring those that are more attractive to honeybees. This shift can reduce the diversity of plant species that rely on native pollinators, potentially altering community composition and function.

However, not all native pollinator species experience the same degree of abundance decline in honeybee-invaded ecosystems. Several factors contribute to this variation. First, differences in ecological niches play a role, as some native pollinators specialize in specific plants or resources that are less preferred by honeybees, thereby reducing competition [9,10,11]. For example, native pollinators that specialize in deep, tubular flowers, are less affected by introduced honeybees, which prefer more accessible flowers [10]. Additionally, native pollinators with different activity patterns, such as those that forage earlier in the morning or later in the evening when honeybees are less active, may experience reduced competition [12]. Second, differences in species abundance influence the extent of impact. Rare pollinators with lower densities are typically more sensitive to the dominant invaders compared to common pollinators with higher densities [13,14]. This is likely because rare pollinators often interact with specific resources, which are often a subset of those utilized by generalist honeybees, leading to intensified competition [15]. Furthermore, the introduced honeybees may alter plant community composition and structure, potentially decreasing the abundance of plants preferred by some rare pollinators [16]. In contrast, common pollinators, with broader ecological niches, are often more adaptable to changes in resource availability caused by introduced honeybees [11]. With this in mind, we can hypothesize that (1) native bee species with greater niche overlap with introduced honeybees would experience greater declines in abundance in the presence of introduced bees, and that (2) rare species with lower densities would experience greater declines in abundance compared to common species with higher densities.

Native pollinators can also adapt to the presence of introduced western honeybees by shifting their ecological niches [17]. For example, native pollinators may reduce direct competition with honeybees by altering their foraging times, diet preferences, or habitat use. Behavioral shifts, such as foraging at different times of the day or focusing on alternative underutilized floral resources, can allow native pollinators to coexist with introduced honeybees [11,18,19]. Over generations, native pollinators that successfully shift their ecological niches may gain a survival advantage, making their populations more resilient to the presence of introduced honeybees. However, previous studies have largely overlooked the importance of niche shifts in shaping native pollinator population dynamics. These studies typically predict changes in native pollinator abundance based on the niche overlap (perceived apparent competition) between introduced honeybees and native pollinators in the introduced areas, rather than considering the niche overlap between introduced honeybees in introduced areas and native pollinators in their native areas [11,16]. This approach may underestimate the niche overlap between introduced and native bees, thereby reducing the ability to predict the invasive honeybee’s impact on native pollinators. Consequently, we can hypothesize that (3) niche overlap between invasive honeybees in introduced areas and native pollinators in their native habitats is a stronger predictor of invasive honeybees’ impact on native pollinators than niche overlap within invaded areas.

In this study, we investigated the abundance response of native bees to the introduced western honeybee in alpine meadows on the eastern Tibetan Plateau, where native bee species diversity is particularly high [20]. We compared the species abundance of 15 native bee species between sites close to beekeeper hives (with high densities of introduced bees) and sites far from beekeeper hives (with low densities of introduced bees). According to the aforementioned hypotheses, we predicted that the abundance decline would be greater in the native bee species with greater niche overlap with introduced honeybees and in the rare species with lower densities compared to their counterparts. Additionally, considering the possibility of niche shift in native bee species in response to the introduced honeybees, we predicted that niche overlap between invasive honeybees in introduced areas and native pollinators in their native areas would better predict the invasive honeybee’s impact on native pollinators compared to niche overlap within the invaded areas.

## 2. Materials and Methods

### 2.1. Study Site and Natural History

This study was conducted in an alpine meadow located on the Qinghai–Tibet Plateau in Sichuan Province, China (32°48′ N, 102°33′ E, 3500 m a.s.l.). The region experiences a typical continental plateau climate, characterized by short, cool summers and autumns, and a long winter. The annual mean air temperature is 1.7 °C, and annual precipitation ranges from 450 mm to 900 mm, with most precipitation occurring during the growing season from late May to September [21].

The study site is dominated by sedges (*Kobresia setchwanensis*, *Carex* spp.), grasses (*Deschampsia caespitosa*, *Festuca ovina* and *Elymus nutans*), and forb species (*Saussurea nigrescens*, *Polygonum viviparum*, *Carum carvi*, *Pedicularis kansuensis* and *Anemone trullifolia* var. *linearis*). The primary vegetation type is an alpine meadow, with total vegetation coverage exceeding 95% and plant height averaging ~30 cm [22].

The grassland serves as a summer or winter pasture. The winter pasture, used as our study site, is grazed by livestock during the winter only. Additionally, the study site experiences intense apiculture during the summer due to the abundance of nectar-rich species, including Asteraceae, Lamiaceae, Boraginaceae, Umbelliferae, and Fabaceae [23].

Since the 1980s, the number of domesticated honeybees (*Apis mellifera*) has increased significantly in Hongyuan County. Honeybees cannot overwinter on the plateau, so beekeepers move hives from other regions to the meadows during the growing season (May to September). It is estimated that there are currently ~300 beekeepers and approximately 80 million honeybees every summer on the winter-grazing pasture, covering about 1500 km^2^ of the county. The estimated annual honey production ranges between 40 and 60 tons [24].

### 2.2. Field Sampling

In 2023, we selected three apiaries as reference sites. Each apiary has been operational for over 30 years, with at least 40 hives per year [24]. We chose three plots near the apiaries and three plots distant from them, each plot covering >1 ha. Apiaries were spaced >2 km apart, with paired experimental plots (nearby vs. distant relative to an apiary) separated by >10 km. *A. mellifera* becomes extremely rare when sites are >6 km from the apiary, no *A. mellifera* was observed or captured in the distant plots during any survey. Furthermore, the plant community was dominated by Asteraceae in each plot.

Field sampling involved the use of light muslin sweep nets (38 cm diameter, 180 cm handles) to survey the abundance of introduced and native bees. We conducted five surveys at approximately two-week intervals for each plot. For each survey, sweeping was performed three times; each sweeping was conducted at a constant speed for 50 nets. All sweepings were carried out in the central area of each plot. Sampling occurred between 11:00 am and 3:00 pm on sunny days. All bees collected in the nets were identified and recorded based on morphological traits. Unidentified species were taken to the laboratory for taxonomic identification.

We calculated the sum and average abundance values across the five surveys for each plot and determined the relative species abundance of native bees for both nearby and distant plots using the average data from the three sites. During the flowering season (late June to early September), we conducted ten surveys at approximately ten-day intervals for each community. For each survey, we collected all flower-visiting bees (Hymenoptera: Apoidea) by walking along three prearranged transects (100 m in length, 2 m in width) within each plot. Surveys were conducted between 9:30 am and 4:30 pm on sunny days. We captured >15 individuals for each bee species in each plot, and these samples were taken to the laboratory for palynological analysis. A total of 656 bee individuals were collected, and 2203 bee individuals were recorded during sweeping. Among native bees in the distant plots, *Andrena tarsata* was the most abundant species (22.54% relative abundance), while *Sphecodes* sp. was the rarest (0.35% relative abundance). Nine bee species had relative abundances below 5%, so they were classified as rare.

In addition, we surveyed the flowering plant community five times with two-week intervals during the flowering season (late June to early September) in each plot. For each survey, we randomly placed twenty-five 1 m × 1 m quadrats in each plot. We recorded the number of flowers or inflorescences for each plant species in each quadrat, and calculated flower abundance for each plant species.

### 2.3. Identification of Pollen Carried by Bee Individuals

For palynological analysis, we sampled pollen grains from all collected bee individuals. Each insect specimen was bathed in 95% ethanol in a centrifuge tube using an ultrasonic cleaner for ten minutes. Each bathed bee was removed and preserved in a second co-referenced tube in 95% alcohol. The pollen samples remaining in the first tube were centrifuged at 5000 rpm for 10 min, and the supernatants were decanted. Each pollen sample was then placed on slides with a micropipette. We identified the pollen contained on the slides at 100 and 400× magnifications using a light microscope (Nikon, E600) by comparing them to a pollen reference library constructed from field-collected and identified flowers.

To construct the pollen library, we collected 95 flowering plant species from the alpine meadow throughout the flowering season. The dehisced anthers from each flower were suspended in 95% ethanol and photographed under a microscope using the same method as described above.

An insect specimen was considered to be a potential pollinator of the plant species if a minimum of 3 grains of the same pollen morphotype were observed. We used a slightly lower number of pollen grains than other authors to define interactions [25], because some plant species may have less pollen on individuals of the same bee species in the interactions that have been identified (e.g., some *Asteraceae*, personal observation by Ruimin An).

### 2.4. Perceived Apparent Competition

The collected individuals were identified to 15 different species, including the introduced A. mellifera, 4 Apidae species, 1 Megachilidae species, 3 Andrenidae species, and 7 Halictidae species (Table A1). Ten native bee species were found in both the nearby and distant plots, and five species were found in the distant plots only. Moreover, among the bee species observed in both nearby and distant plots, 6 species are common, having a higher abundance, whereas 9 species are rare with a lower abundance.

We constructed quantitative pollen transport networks (bee species in rows and plant species in columns) for each plot. The strength of each interaction was calculated as the number of individuals collected carrying pollen of the visited plants [25]. The plant and bee species that had no interaction partners were removed from the pollen transport networks. We combined ten surveys in each plot and constructed 6 networks from these six plots.

We calculated perceived apparent competition (PAC) for each network using the bipartite package [26,27] in R version 4.2.1 (R Core Team, 2022). Perceived apparent competition (PAC) estimates the degree of niche overlap between two species using Müller’s index [28,29]. In this study, we only considered the PAC between *A. mellifera* and each native bee species. For all pairwise comparisons of *A. mellifera* against each of native bee species, we calculated Müller’s index as

(1) $$d_{ij}=\sum_{k}[\frac{\alpha_{ik}}{\sum_{I}\alpha_{iI}}\times \frac{\alpha_{jk}}{\sum_{m}\alpha_{mk}}],$$

where $\alpha_{ik}$ presents the number of interactions between pollinator i and plant k, $\alpha_{il}$ represents the number of interactions by pollinator i across all plants I, $\alpha_{jk}$ represents the number of interactions between pollinator j and plant k, and $\alpha_{mk}$ represents visits to plant k from all pollinators m.

Müller’s index is sensitive to the relative abundance of competing species. Therefore, we used the number of bee specimens involved in constructing the pollen transport networks to adjust the strength of each interaction before we calculated PAC. We calculated the new number of each interaction as

(2) $$N_{xy}=P_{xy}/A_{x},$$

where $A_{x}$ represents the individuals of pollinator x involved in constructing each network, and $P_{xy}$ represents the number of interactions between pollinator x and plant y in each past network. As for the *A. mellifera*, $A_{x}$ represents the average number of individuals involved in constructing three networks. $P_{xy}$ represents the number of interactions between *A. mellifera* and plant y in each network. Both $N_{xy}$ and $P_{xy}$ are the same in six networks.

For PAC calculations between a native bee species and *Apis mellifera*, we first developed a pollination network matrix exclusively comprising these two species. We then applied the second formula to this matrix to calculate interaction strengths under conditions of equal abundances. Finally, these interaction strengths were substituted into the first formula to determine the PAC metric. We calculated two PAC values for each native bee species using the reconstructed interaction frequencies ($N_{xy}$). One is *PAC_C_*, representing the niche overlap between the introduced and native bee species in the nearby plots, and the other is *PAC_D_*, representing the niche overlap between the introduced bee and native bee species in the distant plots.

### 2.5. Data Analysis

All analyses were conducted using R 4.4.1 (R Core Team, 2024). The networks were visualized using the plotweb function from the bipartite R package v.2.16. Prior to modeling, we examined the distributions of the variables.

We determined whether the presence of A. mellifera was associated with changes in native bee abundance using separate generalized linear models (GLMs) fitted with a negative binomial distribution for each native bee species. We included plot type (nearby vs. distant) as a fixed predictor with the abundance of native bee species as the response variable.

We calculated the relative change in abundance for each native bee species as the abundance difference between nearby and distant sites divided by the bee abundance at distant sites. To compare the predictive performance of *PAC_C_* and *PAC_D_* for the relative change in bee abundance, we assessed whether *PAC_C_* or *PAC_D_* was associated with the relative change in bee abundance using separate generalized linear models (GLMs) fitted with a Gaussian distribution for two types (nearby vs. distant) of plots. Moreover, to determine whether rare species are more sensitive to the introduction of western honeybees, we assessed whether the abundance of native bee species in the distant plots was associated with the relative change in bee abundance using generalized linear models (GLM) fitted with a gaussian distribution.

To determine to what extent species abundance and *PAC_D_* affected the abundance change induced by the introduced honeybees, we assessed whether species abundance in the distant plot, *PAC_D_*, and their interactions were associated with the relative change in native bee abundance using generalized linear models (GLM) fitted with a Gaussian distribution. We included bee abundance in distant plots, *PAC_D_*, and their interactions as predictors and the relative change in abundance as a response variable.

To examine whether native bees changed their feeding niches, we conducted a permutational analysis of variances (PERMANOVA) on the Bray–Curtis dissimilarity matrix including the diet preference of the six common species. Bee diet preference was calculated as the proportion of links for each plant to the total number of links. We combined adjacent samples to ensure consistency of variables. The PERMANOVA was run with 999 permutations using the “adonis2” function in the R package vegan v. 2.6–4 [30]. The dissimilarity in native bees’ niches between nearby and distant plots was also computed using non-metric multidimensional scaling (NMDS) with Bray–Curtis distance and the “metaMDS” function in the R package vegan v. 2.6–4 [30].

To further investigate the similarity in flowering plant species composition across six communities, we performed a permutational analysis of variances (PERMANOVA) on the Bray–Curtis dissimilarity matrix including the flower abundance for each species. The PERMANOVA was run with 999 permutations using the “adonis2” function in the R package vegan v. 2.6–4 [30]. Additionally, we assessed the dissimilarity in flowering plant species composition across six communities using non-metric multidimensional scaling (NMDS) with Bray–Curtis distance. The NMDS analysis was carried out using the “metaMDS” function in the R package vegan v. 2.6–4 [30].

## 3. Results

### 3.1. The Plant–Bee Interaction Network

**Figure 1:**
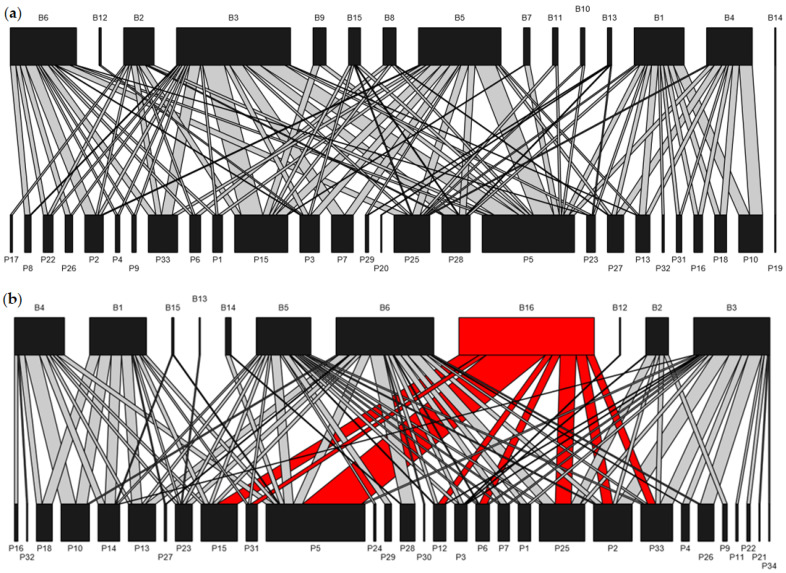
Illustration of a plant–bee network based on the examination of pollen carried by bees in (**a**) distant plots and (**b**) nearby plots. Top nodes represent pollinators, and bottom nodes represent plants. The width is proportional to the frequency of particular pollinators visiting plants. The introduced *A*. *mellifera* (B16) is shown in red. The bee and plant species identities are listed in the Appendix A.

**Figure A1:**
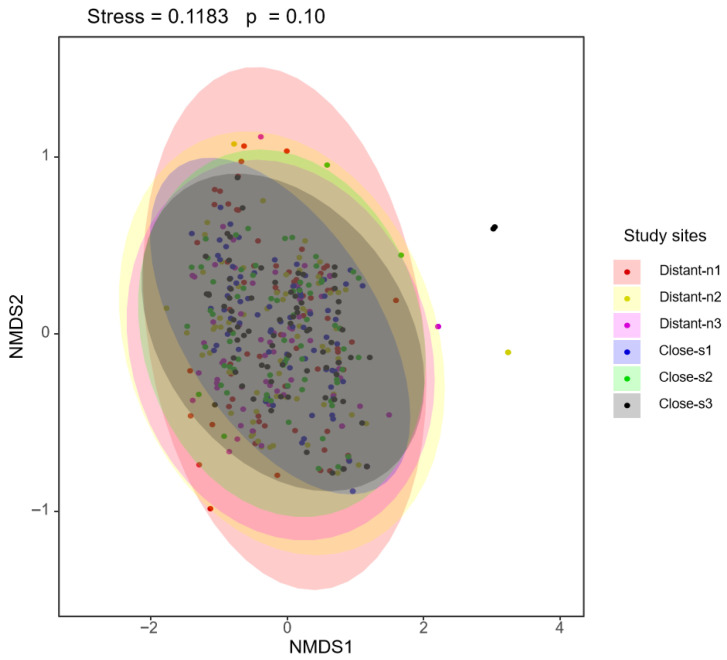
Results of non-metric multidimensional scaling (NMDS) showing plant species community similarity among six field sampling plots. Dissimilarity (Bray–Curtis) was estimated using the abundance of flowers or inflorescences.

Using pollen analysis on the pollen loads carried by the bees, we determined 858 interactions between individual bees and plants. There was a total of 133 species pair interactions in total (Figure 1). Among all native bee species, the most generalist pollinator was *Andrena tarsata*, visiting 22 plant species (41.5% of floral taxa recorded), whereas the extreme specialist *Sphecodes* sp. exclusively foraged on a single plant species (*Angelica dahurica*). Additionally, the PERMANOVA and NMDS analyses indicated no significant differences in flowering plant species composition among the six communities (Figure A1).

### 3.2. The Difference in Native Bee Species Abundance Between Nearby and Distant Plots

**Figure 2:**
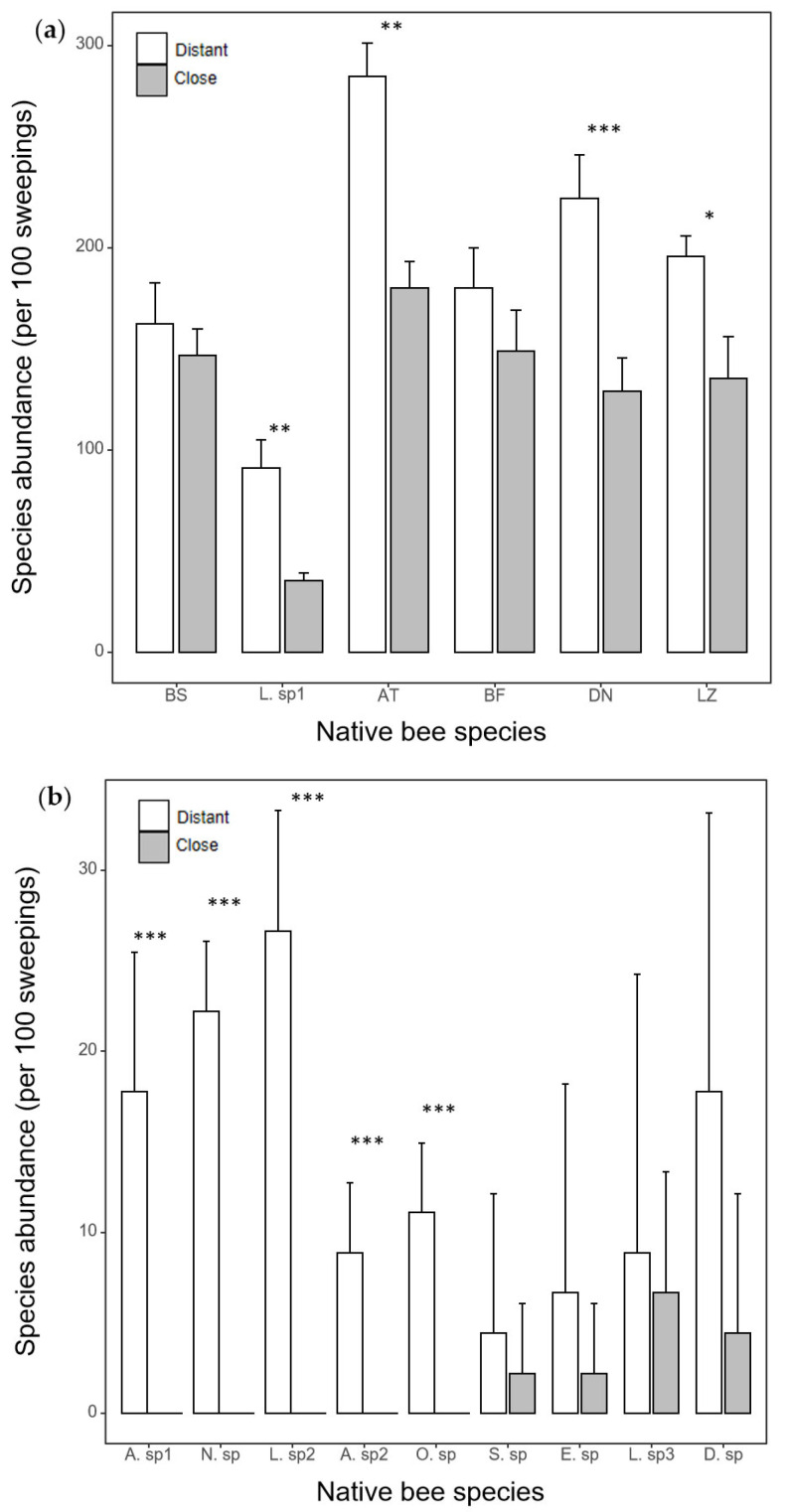
The difference in species abundance of (**a**) 6 common bee species and (**b**) 9 rare species (5 of which were found in the distant plots only) between nearby and distant plots. Error bars represent standard deviation. * *p* < 0.05; ** *p* < 0.01; *** *p* < 0.001. Species names are provided in the Appendix A.

For 6 common species, the presence of *A. mellifera* significantly decreased the abundance of the four species. There was no statistically significant difference in the abundance of the two common species between the nearby and distant plots (Figure 2a). Five rare bee species were found only in distant plots, and the other four bee species remained unchanged by the introduced honeybees (Figure 2b).

### 3.3. PAC and Species Abundance on Relative Change in Bee Abundance

**Figure 3:**
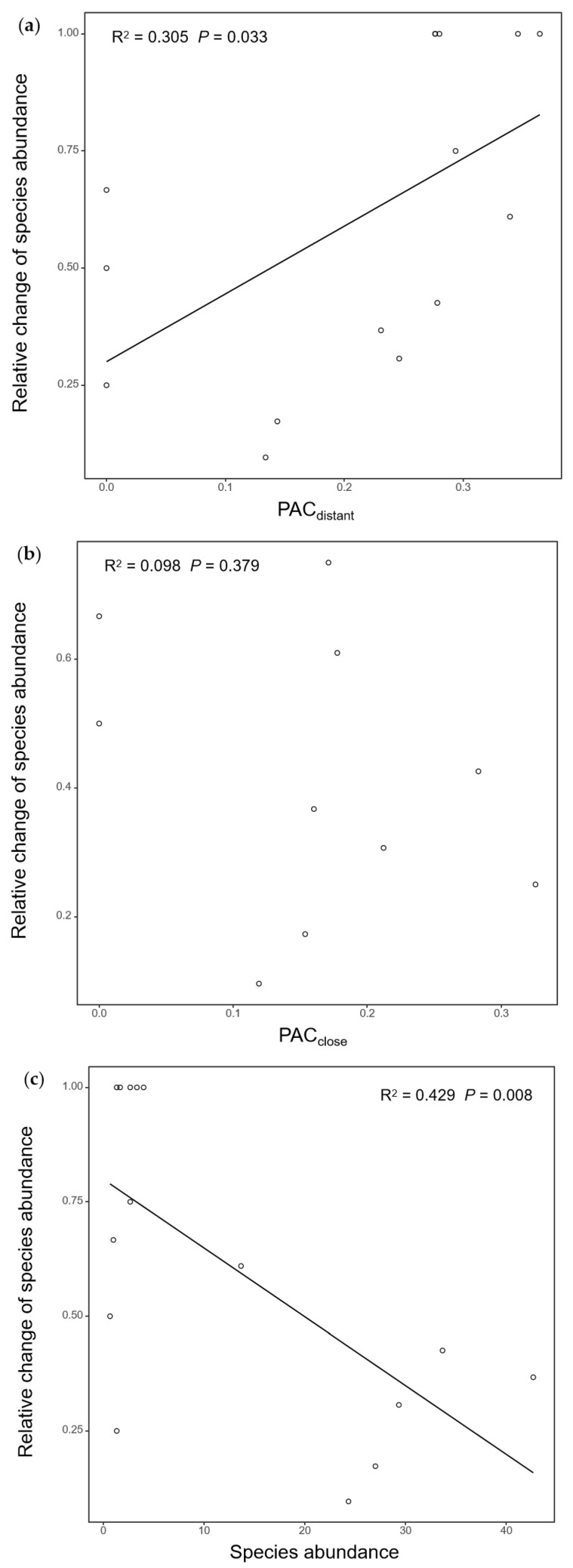
The relationship between relative change in bee abundance vs. (**a**) *PAC_D_*, (**b**) *PAC_C_* and (**c**) bee species abundance in the distant plot across native bee species. N = 15 for both (**a**,**c**); N = 10 for (**b**) because of species loss in the nearby plots.

Across all species that were found in both nearby and distant plots, *PAC_D_* showed a significant positive relationship with the relative change in bee abundance decline (Figure 3a), whereas *PAC_C_* exhibited no significant relationship with the relative change (Figure 3b).

Across all species, species abundance in the distant plots showed a significant negative relationship with the relative change in bee abundance (Figure 3c).

In addition, both species, abundance in the distant plot and *PAC_D_*, significantly affected the relative change in bee abundance, but their interaction effect was non-significant (Table 1).

### 3.4. The Shift in the Feeding Niche of Native Bees

**Figure A2:**
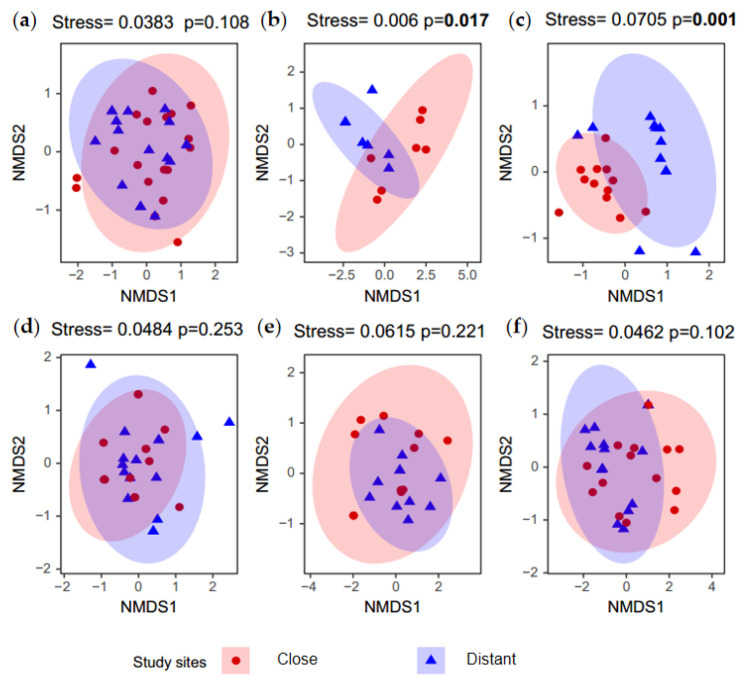
Results of non-metric multidimensional scaling (NMDS) showing whether the feeding niche of native bees differs between nearby (pink) and. distant (gray) plots for six common species. Dissimilarity (Bray–Curtis) was estimated using the diet preference of each sampling. (**a**) *Bombus supremus*, (**b**) *Lasioglossum* sp1, (**c**) *Andrena tarsata*, (**d**) *Bombus filchnerae*, (**e**) *Dufourea novaeangliae*, and (**f**) *Lasioglossum zonulum*. *p*-values are shown in bold as < 0.05.

The PERMANOVA and NMDS analysis showed that significant changes in feeding niche occurred in *Lasioglossum* sp1 and *Andrena tarsata* (Figure A2).

## 4. Discussion

We have shown that among the 15 native bee species we investigated, 9 exhibited significantly lower abundance in distant plots compared to nearby plots where western honeybees (*Apis mellifera*) had been introduced to the alpine meadows of the Tibetan Plateau. Notably, five rare native bee species were not detected in the introduced areas. Given the similarity in flowering plant species composition between nearby and distant plots, our findings suggest that the introduced honeybees had a significantly negative impact on native pollinators in natural ecosystems. The differences in this negative effect among species are likely due to variations in species abundance and the degree of niche overlap between native bees and introduced honeybees. These results highlight the critical importance of understanding ecological interactions involving invasive species, as they can have unintended consequences on local biodiversity.

Floral resources are often a key limiting factor for bee reproduction [31,32,33] and population growth [34,35,36], although parasitism and nest site availability are sometimes more important limiting factors [37,38]. The introduced honeybees have been demonstrated to be highly efficient pollen and nectar foragers and are able to outcompete many native bees by constraining pollen collection and offspring provisioning [39,40]. Consistently, our data show that most of the studied bee species were significantly and negatively impacted by the introduced honeybees. Moreover, some native bees, even if they shift their diet, they still suffer abundance decline, because they are forced to forage on less nutritious plants, spend more time searching for resources that are unoccupied or have not yet been depleted, or foraging further from their nests [11,41,42,43,44,45].

However, the effect of the introduced honeybees was different among native bees. As predicted, perceived apparent competition, a measure of niche overlap, can be used to predict the population dynamics of native bees. This is similar to the finding that the performance of the native generalist megachilid *Osmia pumilia*, which had a higher resource overlap with the introduced honeybees (*A. mellifera*), was more detrimentally affected than other native *Osmia* species having a lower resource overlap [46]. Consistently, in the study, the degree of niche overlap between native bees and the introduced honeybees was positively correlated with the degree of decline of native bee abundance. Specifically, the presence of the *A. mellifera* had no significant effect on the abundance of the two bumble bees, which have the least niche overlap with the introduced honeybees. This is because only bumble bees can utilize the plant species with complex floral morphologies (e.g., tubular, funnel-shaped, and campanulate corollas), as found in temperate, arctic, and alpine zones of the Northern Hemisphere [9,10]. The depth of the floral tube in these plants has been selected to correspond to the proboscis length of bumble bee species, leading to specialized mutualisms with specific bumble bee species [10]. In the current study, they did have a lower PAC with *A. mellifera* compared to other native bees, although bumble bees might have competed with *A. mellifera* by visiting abundant Asteraceae plants (e.g., *Saussurea nigrescens*).

It is worth noting that *PAC_D_*, but not *PAC_C_*, can successfully predict the interspecific difference in abundance decline among different native bee species. This is presumably because some native bees might have shifted their feeding niche in invaded sites, which might broaden their diet to improve their survival and reproduction, or narrow their diet breath to reduce population size. Indeed, the rare honeybee species are also observed to experience diet change in response to the introduced honeybees. For example, the species *Andrena tarsata* became more specialized at sites with *A. mellifera*, and it visited a lower proportion of available plant species in the invaded sites. Similarly, the other species *Lasioglossum* sp1 also showed a reduction in the proportion of links to plants visited primarily by *A. mellifera*, and an increase in the proportion of links to plants not primarily visited by *A. mellifera*, in response to competition for resources by *A. mellifera*. Such a diet shift is similar to those observed in other studies [15,47].

The difference in the effect of introduced honeybees can also be explained by species abundance. As predicted, the abundance of rare species disproportionally more rapid than that of common species. Notably, five endemic species were found to have disappeared in the invaded sites. One potential cause leading to the disappearance is that the rare species occupy similar and little differential feeding niches to superior competitors of common species including the introduced species. Indeed, except for few exclusive floral resources, these rare and endemic species are observed to visit common plant species including *Saussurea nigrescens*, *Allium chrysanthum*, *Taraxacum sikkimense*, *Angelica apaensis*, which are also visited by the introduced honeybees. Consistent with previous studies, rare pollinators with lower densities are typically more sensitive to the dominant invaders compared to common pollinators with higher densities [13,14]. This is likely because rare pollinators often interact with specific resources, which are often a subset of those utilized by generalist honeybees, leading to intensified competition [15]. Once the abundance of these rare species declined due to competition with the generalist introduced honeybees, they likely experienced local species loss rapidly because of environmental and demographic stochasticity [48].

It must be noted that, theoretically, our methodology—comparing nearby and distant plots—cannot exclude the possibility that the observed differences in native bee abundance were due to initial differences in plant and pollinator communities. This is because we lack pre- and post-apiculture data for the same sites [16,49]. However, in the current study, the flowering plant species composition was similar across all plots, and the soil within each plot is classified as meadow soil according to the Chinese soil classification system [50]. Based on these factors, we attribute the observed decline in native bee abundance to the introduced honeybees.

## 5. Conclusions

Our study demonstrates that, among 15 native bee species investigated, 9 species had a significantly lower abundance in distant plots than close plots, where western honeybees (*Apis mellifera*) had been introduced in alpine meadows of the Tibetan Plateau. In particular, five of these nine species were not detected in the introduced areas. Our findings suggest that the introduced honeybees likely outcompete many native bee species, potentially leading to local species loss, particularly among rare native bee species. Importantly, our results indicate that the interspecific difference in the abundance decline among different native pollinator species are well explained by their species abundance and niche overlap with the introduced honeybees. This adds to current knowledge that apiculture may inadvertently introduce superior competitive invasive pollinators, causing ecological damage to local pollinators and even protective species, though it is traditionally recognized to be important to agricultural production and economic development. To this end, we recommend limiting apicultural practice in nature ecosystems, especially where native honeybees are rich and rare.

## Figures and Tables

**Table 1:** Effects of species abundance, *PAC_D_*, and their interaction on the relative change in species abundance for 15 native bees (residual standard error: 0.1575 on 11 degrees of freedom; Adjusted R^2^: 0.778). * *p* < 0.05. N = 15.

Response	Predictor	Estimate	df	*p*
Relative change of species abundance	Species abundance	−0.024	1	0.036 *
	PAC_D_	1.476	1	0.002 *
	Interaction	0.036	1	0.432

## Data Availability

Not applicable.

## Appendix A

**Table A1:** List of native honeybee species and their relative abundance in both close and distant sites. The text in the first column corresponds the species code in Figure 1; the abbreviation corresponds the species code in Figure 2.

ID	Family	Species	Abbreviation
B1	Apidae	*Bombus supremus*	BS
B2	Halictidae	*Lasioglossum* sp1	L. sp1
B3	Andrenidae	*Andrena tarsata*	AT
B4	Apidae	*Bombus filchnerae*	BF
B5	Halictidae	*Dufourea novaeangliae*	DN
B6	Halictidae	*Lasioglossum zonulum*	LZ
B7	Andrenidae	*Andrena* sp1	A. sp1
B8	Apidae	*Nomada* sp.	N. sp.
B9	Halictidae	*Lasioglossum* sp2	L. sp2
B10	Andrenidae	*Andrena* sp2	A. sp2
B11	Megachilidae	*Osmia* sp.	O. sp.
B12	Halictidae	*Sphecodes* sp.	S. sp.
B13	Apidae	*Epeolus* sp.	E. sp.
B14	Halictidae	*Lasioglossum* sp3	L. sp3
B15	Halictidae	*Dufourea* sp.	D. sp.

**Table A2:** List of plant species. The text in the first column corresponds the species code in Figure 1.

ID	Family	Species
P1	Gentianaceae	*Gentiana abaensis*
P2	Ranunculaceae	*Trollius farreri*
P3	Apiaceae	*Angelica dahurica*
P4	Ranunculaceae	*Anemone rivularis*
P5	Asteraceae	*Saussurea nigrescens*
P6	Apiaceae	*Angelica apaensis*
P7	Apiaceae	*Semenovia malcolmii*
P8	Rosaceae	*Potentilla anserina*
P9	Rosaceae	*Potentilla discolor*
P10	Orobanchaceae	*Pedicularis kansuensis*
P11	Apiaceae	*Carum carvi*
P12	Geraniaceae	*Geranium pylzowianum*
P13	Amaryllidaceae	*Allium sikkimense*
P14	Fabaceae	*Oxytropis kansuensis*
P15	Amaryllidaceae	*Allium chrysanthum*
P16	Gentianaceae	*Halenia elliptica*
P17	Gentianaceae	*Comastoma pulmonarium*
P18	Orobanchaceae	*Pedicularis longiflora*
P19	Asteraceae	*Leontopodium souliei*
P20	Rosaceae	*Dasiphora fruticosa*
P21	Rosaceae	*Geum japonicum*
P22	Ranunculaceae	*Ranunculus tanguticus*
P23	Gentianaceae	*Gentiana formosa*
P24	Geraniaceae	*Geranium pratense*
P25	Asteraceae	*Taraxacum sikkimense*
P26	Ranunculaceae	*Anemone trullifolia*
P27	Fabaceae	*Oxytropis glabra*
P28	Asteraceae	*Ajania przewalskii*
P29	Asteraceae	*Anaphalis flavescens*
P30	Asteraceae	*Saussurea stella*
P31	Fabaceae	*Hedysarum tanguticum*
P32	Orobanchaceae	*Pedicularis* sp.
P33	Asteraceae	*Aster alpinus*
P34	Polygonaceae	*Polygonum viviparum*
